# Diversity and Evolution of Salt Tolerance in the Genus *Vigna*

**DOI:** 10.1371/journal.pone.0164711

**Published:** 2016-10-13

**Authors:** Kohtaro Iseki, Yu Takahashi, Chiaki Muto, Ken Naito, Norihiko Tomooka

**Affiliations:** 1 Biological Resources and Post-harvest Division, Japan International Research Center for Agricultural Sciences, Tsukuba, Ibaraki, Japan; 2 Genetic Resources Center, National Agriculture and Food Science Organization, Tsukuba, Ibaraki, Japan; National Bureau of Plant Genetic Resources, INDIA

## Abstract

Breeding salt tolerant plants is difficult without utilizing a diversity of wild crop relatives. Since the genus *Vigna* (family Fabaceae) is comprised of many wild relatives adapted to various environmental conditions, we evaluated the salt tolerance of 69 accessions of this genus, including that of wild and domesticated accessions originating from Asia, Africa, Oceania, and South America. We grew plants under 50 mM and 200 mM NaCl for two weeks and then measured the biomass, relative quantum yield of photosystem II, leaf Na^+^ concentrations, and leaf K^+^ concentrations. The accessions were clustered into four groups: the most tolerant, tolerant, moderately susceptible, and susceptible. From the most tolerant group, we selected six accessions, all of which were wild accessions adapted to coastal environments, as promising sources of salt tolerance because of their consistently high relative shoot biomass and relative quantum yield. Interestingly, variations in leaf Na^+^ concentration were observed between the accessions in the most tolerant group, suggesting different mechanisms were responsible for their salt tolerance. Phylogenetic analysis with nuclear DNA sequences revealed that salt tolerance had evolved independently at least four times in the genus *Vigna*, within a relatively short period. The findings suggested that simple genetic changes in a few genes might have greatly affected salt tolerances. The elucidation of genetic mechanisms of salt tolerances in the selected accessions may contribute to improving the poor salt tolerance in legume crops.

## Introduction

Soil salinity is one of the major factors responsible for losses in agricultural production. Salt-affected lands cover approximately 10% of the global land surface and nearly 50% of the irrigated cropland worldwide [1]. Although screening for salt tolerance has been extensively performed on staple food crops, in order to develop salt tolerant crop varieties [2], few accessions have been identified [3–5]. A major problem is the low diversity of salt tolerance in domesticated accessions, especially in legume crops. In the family Fabaceae, the majority of salt tolerant or halophytic species are limited to shrubs or woody plants [6], which are genetically divergent from crop species, and are not suitable for agricultural use.

One solution to overcome the difficulties associated with salt tolerance breeding is the use of wild crop relatives. Although wild ancestors of crops are expected to have adaptability to unfavorable conditions associated with their original habitats, domesticated crops may have lost such adaptability during the domestication process, given that abiotic stress tolerance is not necessary under protective cultivations. Therefore, the greater diversity in salinity tolerance of wild genetic resources should be harnessed. However, in the majority of cases little attention has been paid to the wild crop relatives because of insufficient genetic and phenotypic information [7], although successful screening and analysis have been performed for some species [8, 9]. Recently, the intensive use of wild relatives for crop improvement has been proposed, with support of plant biotechnology [10]. This trend will promote the exploitation and use of wild crop relatives that have yet to be evaluated.

One good example to demonstrate the potential of wild crop relatives is the genus *Vigna*, family Fabaceae. The genus *Vigna* is comprised of over 100 species, including nine that contain domesticated or semi-domesticated accessions. They are distributed across tropical and subtropical regions, including coastal regions [11]. In a previous study, we identified two salt tolerant accessions (ID 17 and 18 in this study) from wild germplasm, which are cross compatible with azuki bean (*V*. *angularis*), an important *Vigna* crop in East Asia [12]. In another study, genetic analysis on a beach species *V*. *marina* (ID66) identified a strong QTL for salt tolerance [13]. In addition, the majority of the species are diploid, with relatively small genome sizes (500–600 Mb), and are capable of some interspecific crosses [14]. As such, the wild *Vigna* accessions are valuable genetic resources for gene isolation and cross breeding *via* straightforward genetic approaches.

However, to effectively harness the salt tolerance of genus *Vigna*, a systematic evaluation of various accessions across various species is necessary. To date, several salt tolerance mechanisms such as Na^+^ exclusion, increasing antioxidant ability, osmotic regulation, and changes in hydraulic conductance have been studied in *Vigna* species [15–21], but these studies were limited to domestic varieties. We evaluated the salt tolerance in a total of 69 accessions, including wild and domesticated accessions collected from Asia, Africa, Oceania, and South America. We measured plant responses to different intensities of salt stress (50 and 200 mM NaCl) under a high transpiration environment in a greenhouse. The objectives of this study were to not only identify salt tolerant accessions but also estimate the mechanisms of tolerance by measuring photosystem activity, biomass, leaf Na^+^ accumulation, and K^+^/Na^+^ ratios. We also estimated the divergence time of accessions by phylogenetic analysis. Based on the results, we classified the accessions into four groups (the most tolerant, tolerant, moderately susceptible, and susceptible) and selected six wild accessions as promising resources of salt tolerance. We also found that mechanisms of salt tolerance were diverse in the tolerant accessions and that salt tolerance had independently occurred several times during speciation, within a relatively short time. As such, the information on salt tolerance provided here will promote the use of *Vigna* genetic resources for crop improvement, cloning genes of salt tolerance, and understanding salt tolerance evolution.

## Materials and Methods

### Plant materials

Table 1 shows all 69 accessions in the genus *Vigna* used in this study, which included 15 domesticated and 54 wild accessions selected from wide ranges of their original habitats. Eight species contained both domesticated and wild accessions. Accessions in the subgenus *Ceratotropis* (taxonomic sections *Angulares*, *Ceratotropis*, and *Aconitifoliae*) represent the Asian *Vigna*, whereas those in the subgenus *Plectrotropis* (section *Plectrotropis*) and the subgenus *Vigna* (sections *Catiang* and *Vigna*) represent the African *Vigna*. All of the accessions were provided by the Genebank, National Agriculture and Food Research Organization (NARO, formerly called NIAS), Tsukuba, Japan.

**Table 1:** Accessions used in this study.

Subgenus	Section	ID	Species	Types	Origin	Accession number	rDNA-ITS (bp)	DDBJ No.
*Ceratotropis*	*Angulares*	(1)	*V*. *angularis* var. *nipponensis*	Wild	Japan	JP87910	557	LC081993
		(2)	*V*. *angularis* var. *nipponensis*	Wild	Laos	JP226665	557	LC081995
		(3)	*V*. *angularis* var. *angularis*	Domesticated	Japan	JP37752	557	LC081992
		(4)	*V*. *tenuicaulis*	Wild	Myanmar	JP227438	557	LC081991
		(5)	*V*. *nepalensis*	Wild	Nepal	JP107881	557	LC081994
		(6)	*V*. *tenuicaulis*	Wild	Thailand	JP109682	557	LC081990
		(7)	*V*. *umbellata*	Domesticated	Japan	JP99485	557	LC081982
		(8)	*V*. *umbellata*	Wild	Thailand	JP109675	557	LC081981
		(9)	*V*. *umbellata*	Wild	Thailand	JP210639	557	LC064307
		(10)	*V*. *hirtella*	Wild	Thailand	JP109681	557	LC081983
		(11)	*V*. *hirtella*	Wild	Sri Lanka	JP218935	557	LC081984
		(12)	*V*. *exilis*	Wild	Thailand	JP205884	557	LC081985
		(13)	*V*. *hirtella*	Wild	Thailand	JP108562	563	LC081989
		(14)	*V*. *hirtella*	Wild	Laos	JP220137	558	LC081988
		(15)	*V*. *reflexo-pilosa* var. *glabra*	Domesticated	Philippines	JP109684	557	LC081986
		(16)	*V*. *reflexo-pilosa* var. *reflexo-pilosa*	Wild	Malaysia	JP108867	557	LC081987
		(17)	*V*. *nakashimae*	Wild	Japan	JP107879	556	LC082002
		(18)	*V*. *riukiuensis*	Wild	Japan	JP108810	556	LC082001
		(19)	*V*. *minima*	Wild	Indonesia	JP218938	556	LC082000
		(20)	*V*. *minima*	Wild	Papua new guinea	JP226877	556	LC081999
		(21)	*V*. *minima*	Wild	Thailand	JP107869	556	LC081998
		(22)	*V*. *dalzelliana*	Wild	India	JP235419	557	LC081997
		(23)	*V*. *dalzelliana*	Wild	Myanmar	JP210811	557	LC081996
		(24)	*V*. *trinervia*	Wild	Thailand	JP108840	561	LC064352
*Ceratotropis*	*Ceratotropis*	(25)	*V*. *radiata*	Domesticated	Thailand	JP110830	595	LC064348
		(26)	*V*. *radiata* var. *sublobata*	Wild	Madagascar	JP107877	587	LC064349
		(27)	*V*. *radiata* var. *sublobata*	Wild	Papua new guinea	JP226874	597	LC082004
		(28)	*Vigna sp*. (NI1135)	Wild	India	JP110836	564	LC064353
		(29)	*V*. *mungo*	Domesticated	India	JP109668	562	LC064346
		(30)	*V*. *mungo* var. *silvestris*	Wild	India	JP107874	562	LC064347
		(31)	*V*. *sahyadriana*	Wild	India	JP235420	568	LC082003
		(32)	*V*. *grandiflora*	Wild	Thailand	JP107862	562	LC064345
*Ceratotropis*	*Aconitifoliae*	(33)	*V*. *khandalensis*	Wild	India	JP253828	561	LC082005
		(34)	*V*. *subramaniana*	Wild	India	JP229284	562	LC064350
		(35)	*V*. *subramaniana*	Wild	India	JP229278	562	LC064351
		(36)	*V*. *stipulacea*	Wild	Sri Lanka	JP205892	562	LC082006
		(37)	*V*. *stipulacea*	Wild	India	JP245503	561	LC082007
		(38)	*V*. *aconitifolia*	Domesticated	India	JP245897	562	LC082017
		(39)	*V*. *aconitifolia*	Domesticated	Pakistan	JP104332	562	LC082016
		(40)	*V*. *aconitifolia*	Wild	India	JP235416	562	LC082014
		(41)	*V*. *aconitifolia*	Wild	India	JP245865	562	LC082013
		(42)	*V*. *aconitifolia*	Wild	India	JP245864	562	LC082012
		(43)	*V*. *aconitifolia*	Domesticated	India	JP245857	562	LC082015
		(44)	*V*. *indica*	Wild	India	JP235417	562	LC082011
		(45)	*V*. *trilobata*	Wild	Sri Lanka	JP210605	562	LC082009
		(46)	*V*. *trilobata*	Wild	India	JP245881	562	LC082010
		(47)	*V*. *trilobata*	Wild	Sri Lanka	JP205895	562	LC082008
		(48)	*V*. *aridicola*	Wild	Sri Lanka	JP207977	561	LC082020
		(49)	*V*. *aridicola*	Wild	Sri Lanka	JP205896	561	LC082019
		(50)	*V*. *aridicola*	Wild	Sri Lanka	JP205894	561	LC082018
*Plectrotropis*	*Plectrotropis*	(51)	*V*. *vexillata* var. *vexillata*	Wild	Congo	JP235912	563	LC082039
		(52)	*V*. *vexillata* var. *angustifolia*	Wild	Colombia	JP235869	563	LC082038
		(53)	*V*. *vexillata*	Wild	Papua new guinea	JP230747	563	LC082037
		(54)	*V*. *vexillata*	Wild	Surinam	JP202334	563	LC082036
		(55)	*V*. *vexillata* var. *ovata*	Wild	South africa	JP235908	562	LC082033
		(56)	*V*. *vexillata* var. *macrosperma*	Domesticated	Sudan	JP235905	559	LC082034
		(57)	*V*. *vexillata*	Wild	Brasil	JP202337	562	LC082035
		(58)	*V*. *vexillata*	Domesticated	Indonesia	JP235863	560	LC082032
		(59)	*V*. *vexillata* var. *lobatifolia*	Wild	Namibia	JP235903	557	LC082031
*Vigna*	*Catiang*	(60)	*V*. *unguiculata*	Domesticated	Sudan	JP86879	581	LC082028
		(61)	*V*. *unguiculata* subsp. *sesquipedalis*	Domesticated	Sri Lanka	JP81610	581	LC082029
		(62)	*V*. *unguiculata*	Domesticated	Nigeria	JP86801	581	LC082027
		(63)	*V*. *unguiculata*	Domesticated	Sudan	JP86877	581	LC082026
		(64)	*V*. *unguiculata* subsp. *dekindtiana*	Wild	Nigeria	JP89083	575	LC082030
*Vigna*	*Vigna*	(65)	*V*. *luteola*	Wild	Brasil	JP235855	566	LC082023
		(66)	*V*. *marina* subsp. *oblonga*	Wild	Benin	JP233389	567	LC082024
		(67)	*V*. *luteola*	Wild	Australia	JP236246	566	LC082021
		(68)	*V*. *marina*	Wild	Japan	JP235813	569	LC082022
		(69)	*V*. *subterranea*	Domesticated	unknown	JP79992	575	LC082025

### Growth conditions and stress evaluation

Experiments were conducted in a greenhouse in Tsukuba, Japan during two different seasons. Seeds were sown on April 24, 2014 in the first experiment and on August 28, 2014 in the second experiment. The meteorological environments in the greenhouse during the first experiment were 15.4 MJ day^-1^, 31.4°C/22.9°C (day/night) and 22.4 hPa/6.6 hPa (day/night) for the means of solar radiation, temperature and vapor pressure deficit, respectively. In the second experiment these environments were 13.5 MJ day^-1^, 28.4°C/21.1°C (day/night) and 19.3 hPa/2.8 hPa (day/night). All accessions were grown in granular culture soils with high water permeability in a plastic pot of 10 cm height and 5.5 cm diameter. The nutrient composition in the culture soil was N:P_2_O_5_:K = 0.24:3:0.24 g kg^-1^. For each accession, six pots (one plant per pot) were prepared. Salt stress treatments were initiated three weeks after sowing in both experiments. We prepared three pools 1 m wide, 10 m long, and 30 cm deep, which contained water with 0 mM, 50 mM, or 200 mM NaCl. The bottom half of each pot was then immersed in the pool. We set the moderate salt stress at 50 mM NaCl for two weeks according to our previous study in which sensitive accessions severely wilted within two weeks even at 50 mM NaCl concentration [12]. We also set the severe stress condition of 200 mM NaCl for two weeks so that we could evaluate the differences in more tolerant accessions. As biological replication, two pots (two plants) per accession were used for each stress treatment. After the stress treatment, chlorophyll fluorescence, leaf Na^+^ and K^+^ concentrations, and shoot biomass were measured to evaluate the effects of salt stress. The uppermost fully expanded leaves of all plants were measured for chlorophyll fluorescence and leaf Na^+^ and K^+^ concentrations. The dry weight of the remaining shoot of each plant was measured after incubation at 80°C for 48 h.

### Quantum yield of photosystem II

The effective quantum yield of photosystem II (Φ_PSII_) was measured with a chlorophyll fluorometer (Mini-PAM, Walz Gmbh, Effeltrich, Germany), from 10:00 am through 2:00 pm. Steady-state (*F*_*s*_) and maximum (*F’*_*m*_) yields were obtained with a leaf clip holder under irradiance of approximately 1,000 μmol photon m^-2^ s^-1^ PPFD. Φ_PSII_ was calculated according to a previous study [22], in which Φ_PSII_ = (*F’*_*m*_*—F*_*s*_) / *F’*_*m*_.

Because of differences in the meteorological environments, especially in solar radiation and air humidity between the experiments, the absolute values of the quantum yield were variable between the first and second experiments (S1 Appendix). In the second experiment, the solar radiation was lower and air humidity was higher than in the first experiment. Therefore, relative quantum yields (RQYs: the quantum yields in the stressed condition divided by those in the control) were used to evaluate salt tolerance.

### Cluster analysis

Euclidean distances computed from the average relative quantum yield of photosystem II, under the 50 mM NaCl and 200 mM NaCl conditions, were subjected to hierarchical clustering to identify the patterns of salt response in the 69 accessions. Cluster analyses were performed by Ward’s method [23]. To compare all the evaluated traits between the different groups revealed by cluster analysis, analysis of variance (ANOVA) was performed with Tukey’s multiple range test, using the groups and salt treatments as the factors.

### Na^+^ and K^+^ concentrations

Leaf Na^+^ and K^+^ concentrations were determined using a method from a previous study [24] with some modifications. Three leaf pieces of 7 mm diameter were collected from each plant in a 2 ml tube. Following dry weight determination, samples were ground and suspended in 1 ml of 1 M ammonium acetate and shaken for 1 h. Homogenates were then centrifuged for 5 min at 7,000 ×*g*. The supernatant was measured for Na^+^ and K^+^ concentrations with a LAQUAtwin Na^+^ meter (Horiba, Kyoto, Japan) and LAQUAtwin K^+^ meter (Horiba, Kyoto, Japan), respectively, calibrated with standard solutions. The concentrations were then calculated to represent mmol g^-1^ dry weight.

### Phylogenetic analysis

Phylogenetic trees of all 69 accessions were constructed with the sequence data of the internal transcribed spacer (ITS) region of nuclear ribosomal DNA with *Phaseolus vulgaris* (plant accession number: JP219310, sequence DDBJ number: LC082303) as an out-group. For all the accessions, sequence data [25] were downloaded from the DNA data bank of Japan (DDBJ: www.ddbj.nig.ac.jp, see Table 1 for accession numbers). Phylogenetic analysis was performed by the Neighbor-Joining method with 1,000 bootstraps using MEGA 6.0 software [26]. From the sequence data of all accessions, positions containing insertion/deletion and missing data were eliminated, and a total of 463 bases were used as the final dataset. Evolutionary divergence time was calculated, based on the estimated divergence time between *Vigna* and *Phaseolus*, as 8 million years ago [27].

## Results

### Relative quantum yields

**Fig 1:**
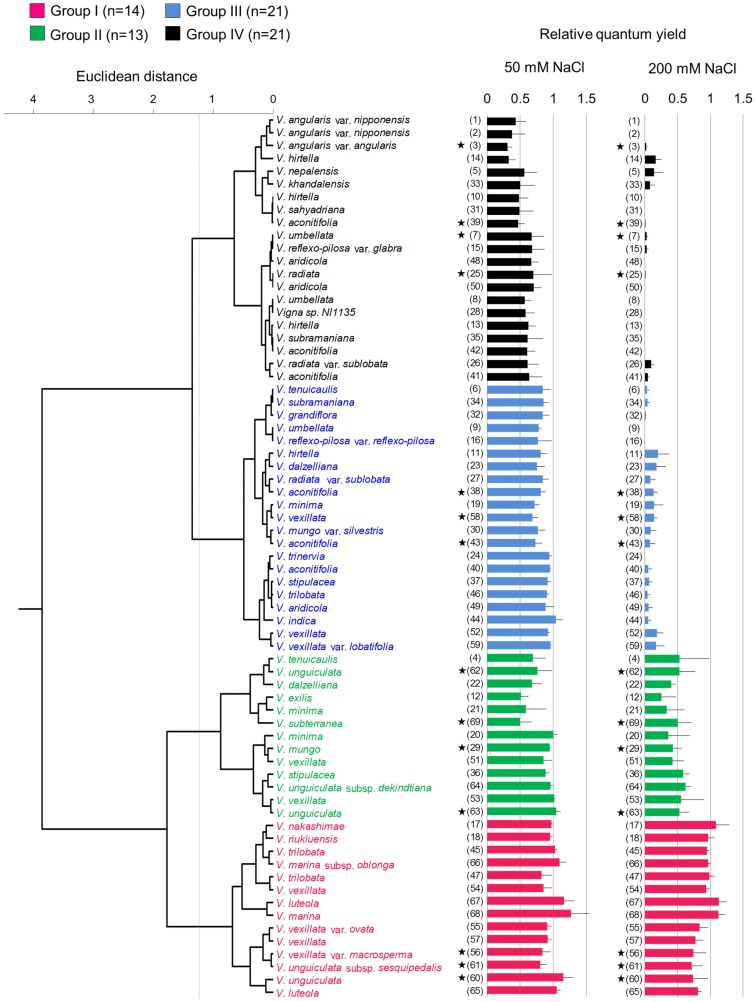
Dendrogram based on cluster analysis on relative quantum yields in salt stress. The four groups identified by cluster analysis are indicated in different colors. Group I: magenta; Group II: green; Group III: blue; and Group IV: black. Bars represent the mean ± standard error (SE) of all plants. ID numbers in the parentheses correspond to those in Table 1. Stars indicate domesticated accessions.

**Fig 2:**
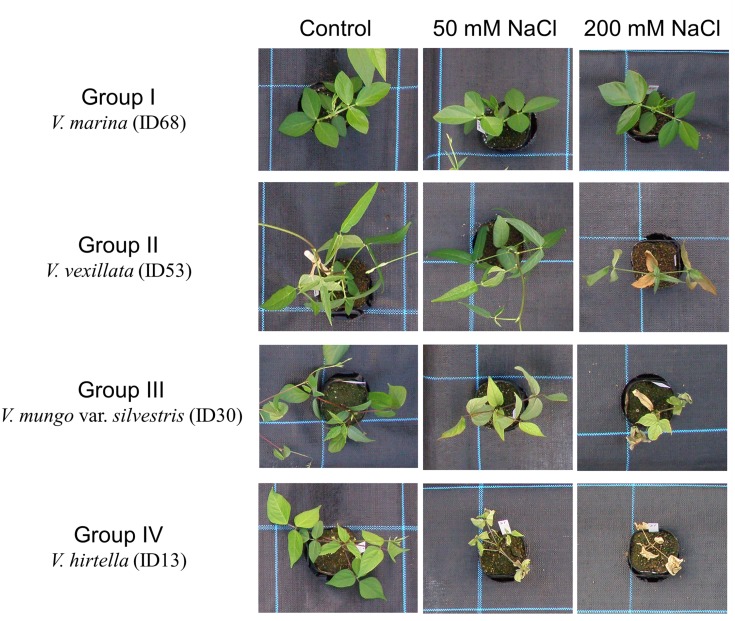
Photos of the accessions representing the four salt tolerant groups. Photos were taken after two weeks of salt stress treatment. Photos of all accession are also available in S1 Fig.

We evaluated salt tolerance of the 69 accessions using RQY (see Materials and Methods); because the quantum yield of photosystem II is commonly used as an indicator of plant response to environmental stresses [28]. The following cluster analysis clearly classified the accessions into four groups (Fig 1). Photos of the representative accessions of each group are shown in Fig 2. The RQY of each accession in each experiment is shown in S1 Appendix.

**Fig 3:**
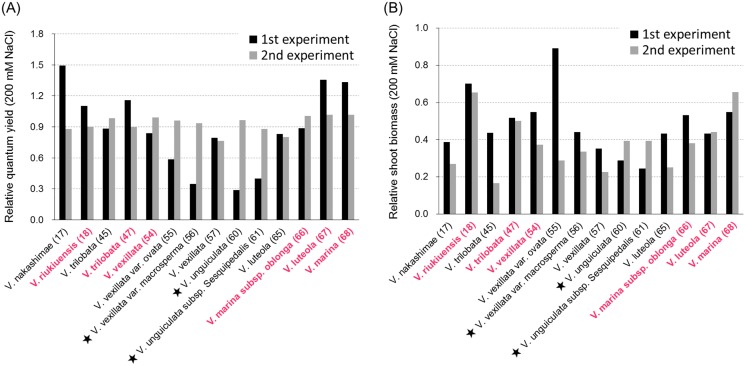
Relative quantum yields and relative shoot biomass of group I accessions in the 1st and 2nd experiments. (A) Relative quantum yields. (B) Relative shoot biomass. Stars indicate domesticated accessions. Accessions in bold red are selected as the most salt-tolerant.

Group I included the 14 accessions that showed the highest RQYs in salt-stressed conditions (Fig 1) and no visual salt damage, even in 200 mM NaCl (Fig 2, S1 Fig). The average RQYs of the accessions were higher than 0.9 in both 50 mM and 200 mM NaCl (Table 2). In this group, RQYs in 200 mM NaCl were repeatedly high in the wild accessions, except *V*. *vexillata* var. *ovata* (ID55) (Fig 3A). In contrast, the RQYs in 200 mM NaCl were not consistent between the experimental replicates in all three domesticated accessions including tuber cowpea (*V*. *vexillata* var. *macrosperma*: ID56), cowpea (*V*. *unguiculata*: ID60), and yardlong bean (*V*. *unguiculata* subsp. *sesquipedalis*: ID61) (Fig 3A). Particularly in the first experiment, the RQYs declined to 0.3–0.4 in these three domesticated accessions. We ranked this group as “the most tolerant.”

**Table 2:** Averages of relative quantum yield, relative shoot biomass, leaf Na^+^ concentration, and K^+^/Na^+^ ratio for each group.

		Relative quantum yield		Relative shoot biomass		Leaf Na^+^ concentration (mmol g^-1^ DW)			K^+^/Na^+^ ratio		
		50 mM	200 mM	50 mM	200 mM	0 mM	50 mM	200 mM	0 mM	50 mM	200 mM
Average	Group I	0.99 a	0.91 a	0.83 a	0.43 a	0.12 a	0.34 b	3.30 a	1.3 a	0.9 a	0.1 ab
	Group II	0.80 b	0.47 b	0.59 b	0.28 b	0.10 a	0.53 ab	2.92 ab	1.2 a	1.2 a	0.2 a
	Group III	0.84 b	0.08 c	0.60 b	0.26 b	0.12 a	0.91 a	2.78 ab	1.1 a	0.4 a	0.1 b
	Group IV	0.55 c	0.03 c	0.48 b	0.25 b	0.12 a	0.91 a	2.21 b	1.2 a	0.4 a	0.1 ab
ANOVA	Treatment	**		**		**			**		
	Group	**		*		ns			ns		
	Treatment × Group	**		ns		**			ns		

Different letters in each column indicate that the values are significantly different at p < 0.05 by Tukey’s multiple range test.

*, **, and ns in the ANOVA table indicate significance at *p* < 0.05, significance at *p* < 0.01, and not significant, respectively.

Group II included 13 accessions, where the average RQY was 0.80 in 50 mM NaCl and 0.47 in 200 mM NaCl (Table 2). Leaves became slightly yellowish in 50 mM NaCl and partially wilted, except *V*. *vexillata* (ID51 and ID53) (Fig 2, S1 Fig). This group included four domesticated accessions such as black gram (*V*. *mungo*; ID29), cowpea (ID62 and ID63), and bambara groundnut (*V*. *subterranea*; ID69). We ranked this group as “tolerant.”

Group III included 21 accessions, where the average RQY (0.84) and visual leaf damages were similar to those of Group II in 50 mM NaCl (Table 2, Fig 2, S1 Fig). In 200 mM NaCl, however, the RQY severely decreased (0.08 in average) and many leaves were wilted (Table 2, Fig 2, S1 Fig). We ranked this group as “moderately susceptible.”

Group IV included the remaining 21 accessions, where the average RQY decreased to 0.55 in 50 mM NaCl (Table 2). The leaves of these accessions became yellowish and partially wilted in 50 mM NaCl and completely wilted under 200 mM NaCl (Fig 2). We ranked this group as “susceptible.”

### Shoot biomass

**Fig 4:**
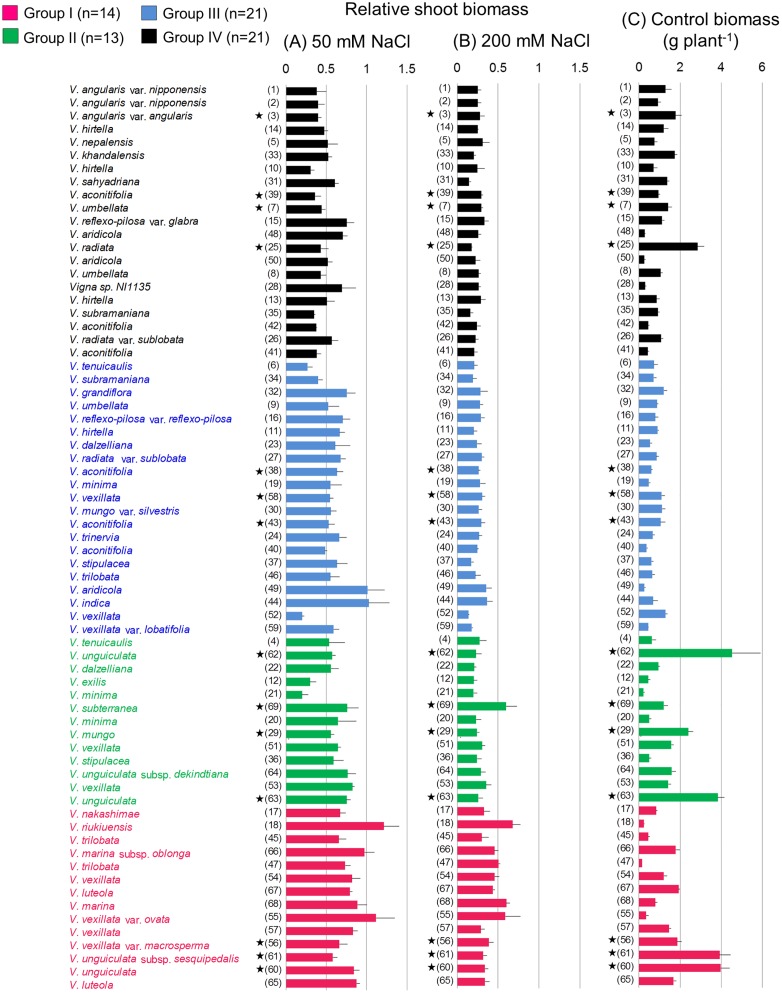
Relative shoot biomass in stressed conditions and absolute shoot biomass. (A) Relative shoot biomass in 50 mM NaCl. (B) Relative shoot biomass in 200 mM NaCl. (C) Absolute shoot biomass in the control condition. Bars represent the mean ± SE of all replicates. Numbers indicate ID numbers. Stars indicate domesticated accessions.

After two weeks of salt treatment, we collected plant shoots and measured the dry weight. Since the absolute shoot biomass in the control condition largely differed between the accessions (Fig 4C), the effects of salt stresses were evaluated according to their relative values (Fig 4A and 4B). As expected, the effect of salt stress on RSB was much more severe in 200 mM NaCl than in 50 mM. Interestingly, many accessions with large shoot biomass were categorized in Groups I and II (Fig 4C).

Although the overall variation in RSB was smaller compared to RQY, the average RSB of Group I was significantly higher than other groups in both 50 mM and 200 mM NaCl (Table 2). In 50 mM NaCl, many accessions in this group showed RSBs of almost 0.9 and the average was 0.83 (Fig 4A, Table 2). In this salt condition, *V*. *riukiuensis* (ID18) grew better than in the control (Fig 4A). In 200 mM NaCl, however, the variation was larger and ranged from 0.27 to 0.68. RSBs were repeatedly high in *V*. *riukiuensis* (ID18), *V*. *trilobata* (ID47), *V*. *vexillata* (ID54), *V*. *marina* subsp. *oblonga* (ID66), *V*. *luteola* (ID67), and *V*. *marina* (ID68), with *V*. *riukiuensis* (ID18) being the highest again (Figs 3B and 4B). In contrast, RSBs fell below 0.3 in at least one experiment in *V*. *nakashimae* (ID17), *V*. *trilobata* (ID45), *V*. *vexillata* var. *ovata* (ID55), *V*. *vexillata* var. *macrosperma* (ID56), *V*. *vexillata* (ID57), *V*. *unguiculata* (ID60), *V*. *unguiculata* subsp. *sesquipedalis* (ID61) and *V*. *luteola* (ID65) (Fig 3B).

In Groups II-IV, the average RSBs were not significantly different from each other (Table 2). However, the RSBs of some accessions in Groups II and III were comparable to those of Group I accessions. In particular, *V*. *subterranea* (ID69), a domesticated accession in Group II, showed the third highest RSB, even exceeding most of the accessions in Group I (Fig 4B). In 50 mM NaCl, accessions of *V*. *indica* (ID44) and *V*. *aridicola* (ID49) in Group III showed particularly high RSBs (Fig 4A).

### Leaf Na^+^ concentration

**Fig 5:**
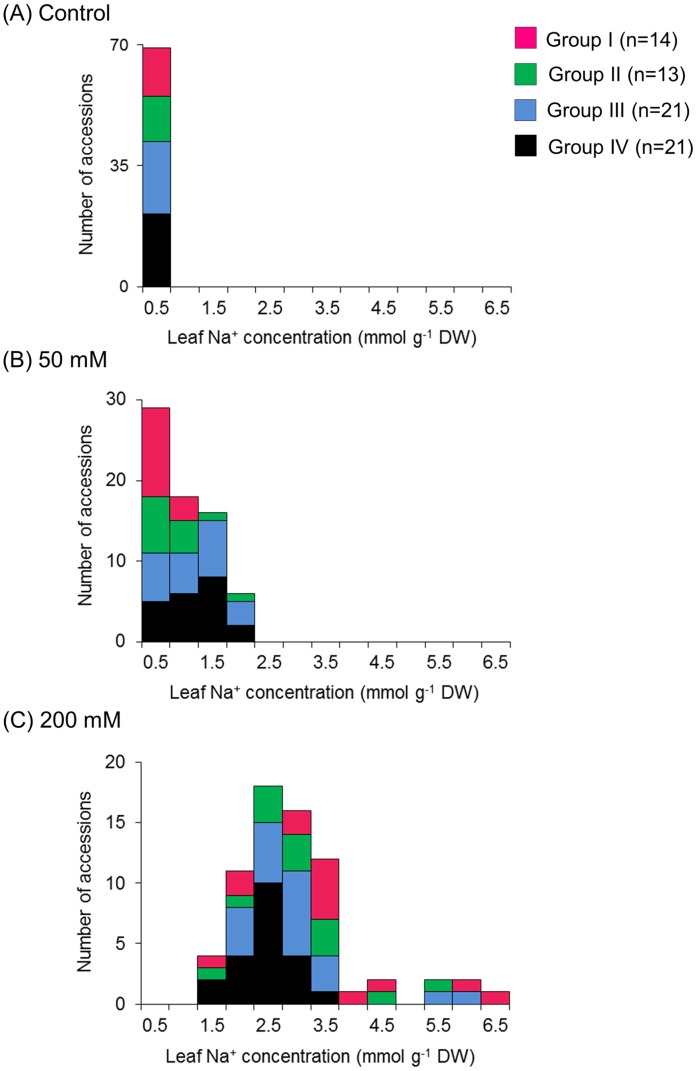
Distribution of leaf Na^+^ concentrations. Distribution of the leaf Na^+^ concentrations in (A) control, (B) 50 mM NaCl, and (C) 200 mM NaCl. Colors represent salt tolerance shown in Fig 1. Group I: magenta; Group II: green; Group III: blue; and Group IV: black.

Plant salt tolerance is considered to involve either avoidance (excluder type) or tolerance (includer type) mechanisms [15]. The “excluder type” plants keeps Na^+^ ions out of internal tissues, whereas “includer type” plants isolate Na^+^ ions in vacuoles [15]. To determine whether the accessions were excluders or includers, we measured Na^+^ concentrations in the topmost fully expanded leaves. In the control condition, leaf Na^+^ concentrations were less than 0.5 mmol g^-1^ in all accessions (Fig 5A) and were not significantly different between the groups (Table 2).

In 50 mM NaCl, Group I accessions accumulated lower amounts of Na^+^ in their leaves, not exceeding 0.8 mmol g^-1^ DW (Fig 5B). In Group II, similar to Group I, most of the accessions showed Na^+^ concentrations < 1.0 mmol g^-1^ DW (Fig 5B). However, some accessions (*V*. *mungo*: ID29 and *V*. *stipulacea*: ID36) exceeded 1.0 mmol g^-1^ DW (S1 Appendix). In Groups III and IV, Na^+^ concentration were highly varied, ranging from 0.1 to 2.0 mmol g^-1^ DW (S1 Appendix), but the averages were significantly higher than Group I averages (Table 2).

In 200 mM NaCl, Na^+^ concentrations increased in all groups, and variations between the accessions became much larger (Fig 5C). In Group I, *V*. *nakashimae* (ID17), *V*. *vexillata* (ID54) and *V*. *luteola* (ID67) maintained leaf Na^+^ concentrations < 2.0 mmol g^-1^ DW (S1 Appendix). In contrast, *V*. *trilobata* (ID47) and *V*. *vexillata* var. *ovata* (ID55) showed the highest concentrations of leaf Na^+^ among all the accessions (5.6 and 6.3 mmol g^-1^ DW, respectively) (S1 Appendix) without displaying symptoms of salt damage (S1 Fig). In Groups II and III, Na^+^ concentrations were also varied, ranging from 1.2 to 5.8 mmol g^-1^ DW. Within these groups, Na^+^ concentrations were higher in *V*. *unguiculata* (ID63) (5.28 mmol g^-1^) and *V*. *vexillata* var. *lobatifolia* (ID59) (5.83 mmol g^-1^), while lower in *V*. *vexillata* (ID53) (1.20 mmol g^-1^) and *V*. *minima* (ID19) (1.50 mmol g^-1^). In Group IV, Na^+^ concentrations were lower than 3.0 mmol g^-1^ DW in their leaves that were already wilted.

### Potassium/sodium ratio

We also measured leaf K^+^ concentration to calculate the K^+^/Na^+^ ratio, which is often used as an important indicator of salt tolerance [29].

**Fig 6:**
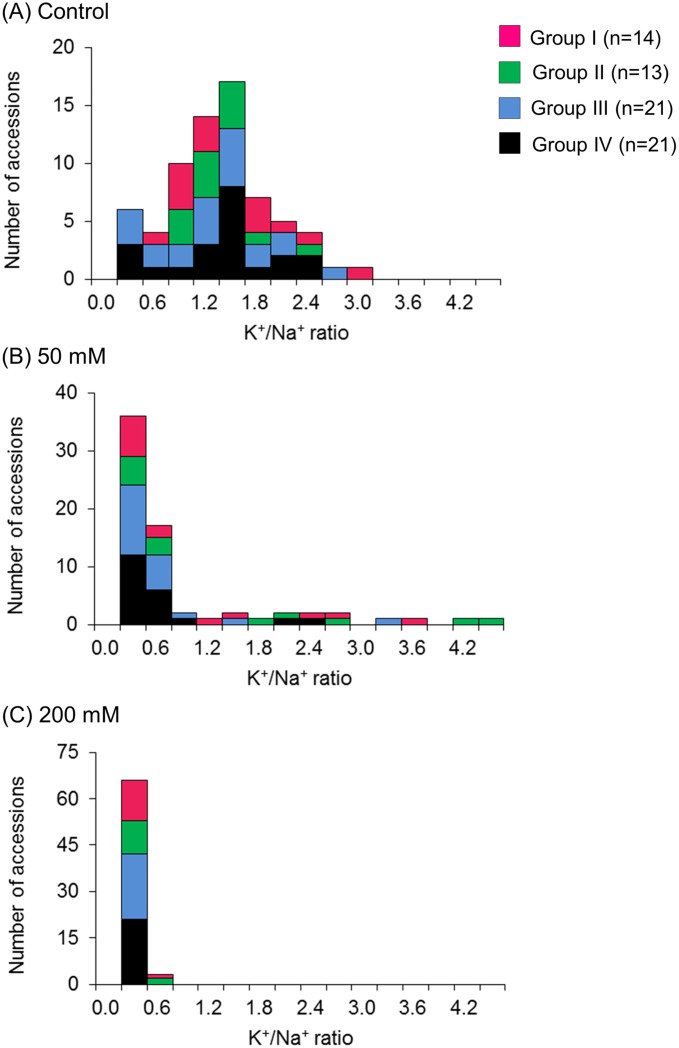
Distribution of K^+^/Na^+^ ratios. Distribution of the leaf K^+^/Na^+^ ratios in (A) control, (B) 50 mM NaCl, and (C) 200 mM NaCl. Colors represent salt tolerance shown in Fig 1. Group I: magenta; Group II: green; Group III: blue; and Group IV: black.

In the control condition, K^+^/Na^+^ ratios varied little between both the accessions and the groups (Fig 6A). In 50 mM NaCl, K^+^/Na^+^ ratios were reduced to < 0.6 in many accessions (Fig 6B). Most accessions with higher K^+^/Na^+^ ratios belonged to the Groups I or II, but two accessions (*V*. *nepalensis*: ID5 and *V*. *hirtella*: ID14) belonged to Group IV (S1 Appendix). In 200 mM NaCl, no accessions showed K^+^/Na^+^ values > 0.6 (Fig 6C).

### Geographical distribution of accessions

**Fig 7:**
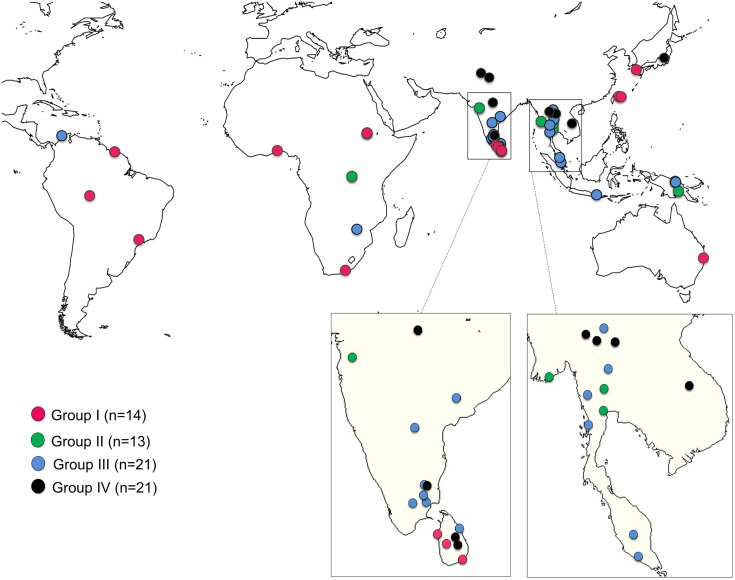
Geographical distribution of accessions. The collection sites of 52 accessions are shown. Colors represent the groups of different salt tolerance shown in Fig 1. Geographical maps were made with Natural Earth free vector and raster map data (http://www.naturalearthdata.com).

**Fig 8:**
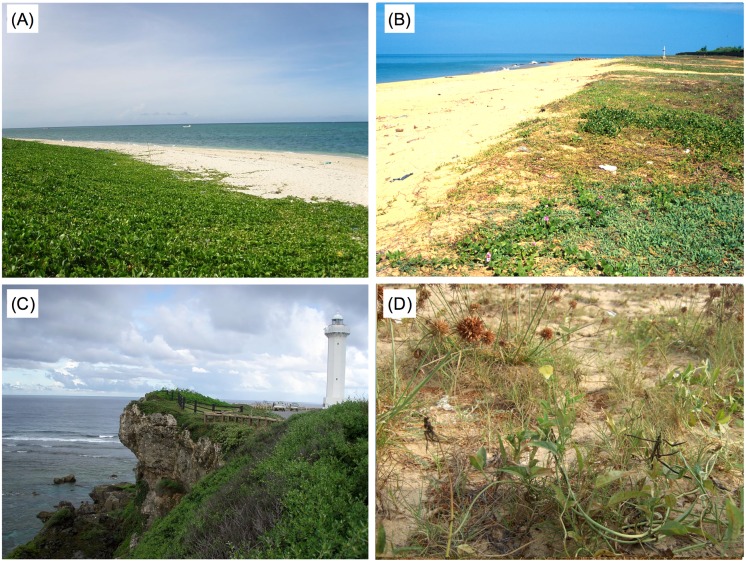
The original habitat of salt tolerant accessions. (A) *Vigna marina* (ID68) in Miyakojima Island, Japan. (B) *V*. *trilobata* (ID47) in Talawila, Northwest Province, Sri Lanka. (C) *V*. *riukiuensis* (ID28) in Miyakojima Island, Japan. (D) *V*. *marina* subsp. *oblonga* (ID66) in Cotonou, Benin.

Of the 69 accessions, we were able to locate collection sites of 52 (Fig 7). Accessions of Groups I and II were mainly collected in coastal or near-coastal areas such as beaches (Fig 8A, 8B and 8D), and coastal cliffs (Fig 8C). Exceptions to this were *V*. *vexillata* var. *vexillata* (ID51), *V*. *unguiculata* (ID60) and *V*. *luteola* (ID65) which originated from inland areas of Africa. Accessions of Group III were mainly found in inland areas, although some of them were found in coastal areas. Accessions of Group IV were all found in inland areas. Accessions of Groups I–III were widely distributed across Asia, Africa, Oceania, and South America. In contrast, accessions of group IV originated only in Asia.

### Divergence time

**Fig 9:**
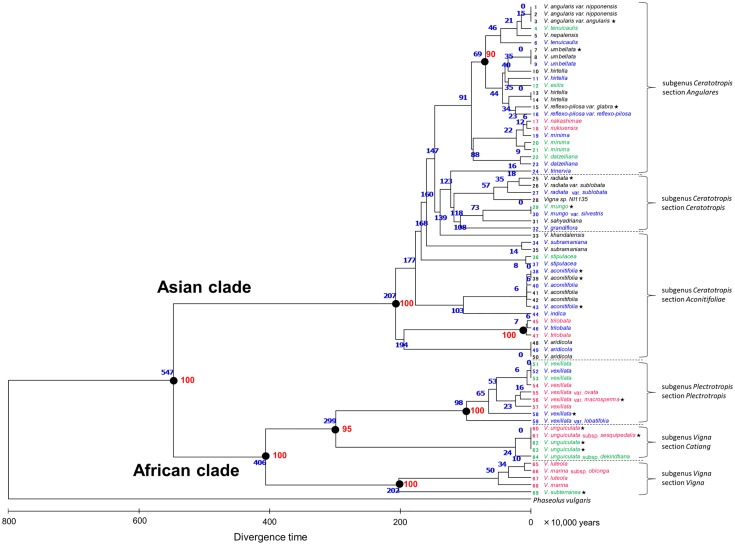
Neighbor-joining tree of the 69 accessions in the genus *Vigna*. The optimal tree with the sum of branch length = 1.35 is shown. Red numbers besides branches indicate bootstrap values (%) with 1,000 replicates. The scale indicates the divergence time in million years. Blue numbers beside branches represent divergence time in 10,000 years. Names of accessions are color-coded to correspond with the four groups shown in Fig 1. Group I: magenta; Group II: green; Group III: blue; and Group IV: black. Rectangles indicate accessions selected as the most salt-tolerant.

To reveal the evolutionary origins of salt tolerance in Group I accessions, we constructed a phylogenetic tree from rDNA-ITS sequences and estimated divergence time of accessions [27]. The phylogenetic relationship agreed with the relationship described in Takahashi et al. [25]. As shown in Fig 9, we color-coded each accession to indicate the group of salt tolerance (see Fig 1). The phylogenetic tree clearly shows that the African accessions (subgenus *Vigna* and *Plectrotropis*) were often in the tolerant groups (I or II) whereas Asian accessions were mostly in the susceptible groups (III or IV).

More importantly, however, the accessions of Group I were present in all taxonomic sections, except section *Ceratotropis*. In addition, the tolerant accessions (Groups I or II) often neighbored susceptible accessions (Groups III or IV). One typical example was the case of *V*. *nakashimae* (ID17), *V*. *riukiuensis* (ID18) and *V*. *minima* (ID19), where the former two (tolerant) had diverged from the latter (susceptible) about 120,000 years ago. Another example was the case of *V*. *trilobata* (ID46 and ID47), where the tolerant accession diverged from the susceptible accession only approximately 60,000 years ago (Fig 9). *V*. *vexillata* (ID54) also diverged from the common ancestor of other accessions (ID51-53) about 60,000 years ago.

## Discussion

In this study, we obtained phenotypic information of several wild *Vigna* accessions regarding salt tolerance. With the information gathered, we were able to identify several salt-tolerant accessions and elucidate the evolutionary origins of salt tolerance.

### Salt tolerant accessions in the genus *Vigna*

Here we selected six accessions as the most salt-tolerant accessions. The selected accessions were *V*. *riukiuensis* (ID18), *V*. *trilobata* (ID47), *V*. *vexillata* (ID54), *V*. *marina* subsp. *oblonga* (ID66), *V*. *luteola* (ID67), and *V*. *marina* (ID68), because they showed higher RQYs and RSBs in both the first and second experiments (Fig 3). Unlike in our previous study [12], we discarded *V*. *nakashimae* (ID17), which we had previously selected as one of the most tolerant accessions in Asian *Vigna* [12]. In the last study, we screened only for visual salt damage whereas, in this study, we considered the RSB. Here *V*. *nakashimae* (ID17) was consistently high in the RQY (Fig 3A), which strongly correlated with visual damage (Fig 2, S1 Fig), but not in the RSB (Figs 3 and 4).

We consider that these accessions could serve as promising sources of salt tolerance for breeding purposes, because, at least some of them are cross compatible with domesticated species. Cross compatible examples are; *V*. *riukiuensis* (ID 18) × azuki bean (*V*. *angularis* var. *angularis*) and *V*. *vexillata* (ID 54) × tuber cowpea (*V*. *vexillata* var. *macrosperma*) [30]. Even wider hybridization is also possible if assisted with embryo rescue [31].

In addition, these accessions could also serve as genetic materials to clone genes involved in salt tolerance. *V*. *trilobata* is the best example of this, as it has an intraspecific variation in salt tolerance (Figs 1 and 9). If we cross ID47 and ID46 of this species, we will be able to develop a population with minimal genetic divergence, except in salt tolerance. In another of our previous studies [13], we demonstrated the strength of this strategy using a mapping population derived from *V*. *marina* subsp. *oblonga* (ID66) and *V*. *luteola* (ID65). Although this study ranked *V*. *luteola* (ID65) in Group I (Fig 1), it does not survive in 350 mM NaCl while *V*. *marina* subsp. *oblonga* (ID66) does not show any symptoms of wilting [13]. Using this approach, we detected a strong QTL that could solely explain 50% of phenotypic variance between the two [13].

Moreover, since the selected accessions are already well adapted to salt-affected environments, it would be interesting to *de novo* domesticate them into new crops, as we recently proposed [32].

### Mechanisms of salt tolerance

We observed two interesting features in the accessions of Group I. First, in 50 mM NaCl, all the accessions accumulated lower amounts of Na^+^ than others (Fig 5B). Second, in 200 mM NaCl, they varied so greatly that the range of leaf Na^+^ concentrations encompassed the lowest to the highest among the 69 accessions (Fig 5C). The first feature indicated that Group I accessions had better salt excluding ability than accessions in other groups. However, the second feature indicated they acquired different strategies to tolerate the severer salt stress. That is, the non-salt-accumulating accessions might be specialized to exclude salt, whereas the salt-accumulating accessions might have added salt-including abilities to isolate excessive Na^+^ ions into vacuoles [29].

This argument agreed with our previous study, where we found *V*. *nakashimae* (ID17) and *V*. *riukiuensis* (ID18) to have different mechanisms of salt tolerance [12]. In that study, we showed the former prevented Na^+^ uptake into leaves, whereas, the latter accumulated high volumes of Na^+^ into leaves. In this study we found the same trend in ID17 and ID18 (1.1 mmol g^-1^ DW vs. 2.8 mmol g^-1^ DW) (S1 Appendix), further confirming the acquisition of different mechanisms of salt tolerance.

Given some of the includer-type accessions and excluder-type accessions are cross-compatible with each other, it may be possible to obtain a plant that is proficient in both abilities. It would be interesting to test whether such a plant could be even more tolerant against salt stress.

On the other hand, though K^+^/Na^+^ ratio is often used as an important indicator of salt tolerance [29], we could not detect a sign of its contribution to salt tolerance in the genus *Vigna*. Several accessions showed higher K^+^/Na^+^ ratios in 50 mM NaCl, but only *V*. *nakashimae* (ID 17) and *V*. *vexillata* (ID 53) retained high RQY (S1 Appendix). In addition, accessions with low leaf Na^+^ concentrations did not always maintain K^+^/Na^+^ ratios. In 200 mM NaCl, no accessions maintained K^+^/Na^+^ ratios (Fig 6). As such, we concluded that K^+^/Na^+^ ratio is not a useful indicator for salt tolerance in the genus *Vigna*. This has also been reported as true for several subspecies of tetraploid wheat [33].

### Evolution of salt tolerance

According to our evaluation of salt tolerance and phylogenetic analysis, we argued that the evolution of salt tolerance, in the genus *Vigna*, has independently occurred several times. Although the selected accessions were all wild accessions adapted to coastal environments (Figs 7 and 8), the phylogenetic tree indicated multiple evolutionary origins of salt tolerance. *V*. *riukiuensis* (ID18) belonging to the section *Angulares*, where most accessions were grouped into Groups III or IV. It had probably acquired salt tolerance after divergence from the ancestor of *V*. *minima* (ID19). Likewise, *V*. *trilobata* (ID47) had acquired salt tolerance after divergence from the ancestor of *V*. *aridicola* (ID41-43). For *V*. *vexillata* (ID54), salt tolerance originated after diverging from the susceptible accessions (ID51-53). The origin of salt tolerance in the accessions of *V*. *marina* and *V*. *luteola* was probably after the divergence from the section *Catiang*. As such, extreme salt tolerance has independently evolved at least four times in the genus *Vigna*. In addition, the phylogenetic positions of other Group I accessions suggested that salt tolerance could have evolved even more frequently. A good example is, again, the case of *V*. *nakashimae* (ID17) and *V*. *riukiuensis* (ID18). Since these two accessions are, as mentioned in the above section, different in their mechanisms of salt tolerance [12], the evolution of salt tolerance should have occurred after divergence of the two species (60,000 years ago) (Fig 9). Similarly, the origin of salt tolerance in *V*. *vexillata* (ID55-57) might be independent from that in ID54 because they formed a different clade (Fig 9). If we take Group II accessions into consideration, we could even postulate that salt tolerance could “often” evolve in the genus *Vigna*.

Moreover, we should note that many tolerant accessions diverged from the susceptible accessions only 50,000–200,000 years ago (Fig 9), indicating the salt tolerance can evolve within a relatively short time. As such, we expect salt tolerance in the genus *Vigna* might have occurred *via* simple genetic changes with large effects. This hypothesis is again supported by our previously study, where we found a strong QTL of salt tolerance from *V*. *marina* subsp. *oblonga* (ID66) [13].

### Salt tolerance in *Vigna* crops

Our results showed that some *Vigna* crops were tolerant to salt stress, such as cowpea, bambara groundnut, tuber cowpea, and black gram. Of these crops, all were African *Vigna*, except black gram. Most of the Asian *Vigna* crops (azuki bean, rice bean, mung bean, and moth bean) were susceptible. The tolerant crops might be directly applied to cultivation in moderately saline soils.

The results agreed with the data shown in the Annex 1 (crop salt tolerance data) in FAO Corporate Document Repository, except black gram (*V*. *mungo*; ID29). In the Annex 1 Table, cowpea was classified as “Moderately Tolerant” while mung bean and black gram were categorized as “Susceptible,” based on shoot biomass and seed yield under long-term salt stress [34].

Unfortunately, we tested only one accession of wild cowpea (*V*. *unguiculata* var. *dekindtiana*; ID64), which was not more tolerant to salt stress than the domesticated cowpea. However, we expect there will be wild cowpea accessions with higher salt tolerance, given the broad intraspecific variation in cowpea varieties [35]. With such materials, it would be possible to further improve salt tolerance in domesticated cowpea.

In the section *Angulares*, none of the domesticated accessions and few of the wild accessions were tolerant to salt stress. As such, to improve the salt tolerance of azuki bean and rice bean, we might have no choice but to cross them with *V*. *nakashimae* (ID17) or *V*. *riukiuensis* (ID 18).

### Salt tolerance vs. absolute shoot biomass

Since trade-offs between stress tolerance and growth rate have been reported [36], we expected salt tolerance would be found only in small accessions. This was true in our previous study, where we screened Asian *Vigna* accessions for salt tolerance and found only small accessions were tolerant against salt stress [12].

However, the results in this study suggested the salt tolerance might not severely affect growth. Firstly, Group I included accessions with the largest shoot biomass (ID60 and ID61) (Figs 1 and 4). This group also included accessions with relatively larger biomasses, such as *V*. *vexillata* var. *macrosperma* (ID56), *V*. *luteola* (ID 65 and ID67) and *V*. *marina* subsp. *oblonga* (ID66). Although the highest RSB was observed in one of the smallest accession (*V*. *riukiuensis* (ID18)), much larger accessions (*V*. *marina* (ID68) and *V*. *subterranea* (ID69)) showed almost the same RSBs in 200 mM NaCl (Fig 4). Moreover, the absolute biomass did not negatively correlate with salt tolerance, even between accessions with closer genetic distances, for example, within *V*. *mungo* (ID29 vs. ID30) or within *V*. *unguiculata* (ID60 and ID61 vs. ID62 and ID63). As such, the cost for salt tolerance may not be as destructive to plant growth as we had expected, or might even have little effect. We now consider that it would be possible to improve salt tolerance of crops without severely reducing growth rate.

However, we should be aware that we treated the plants with salt stress only for two weeks. This was because we had to evaluate the plants before the susceptible accessions had died. However, currently we have no information about grain yield of the tolerant accessions under salt stress. As such, it would be very important to evaluate the accessions in Groups I and II over a longer time period, until seed setting.

## Conclusions

In this study, we evaluated several aspects of salt tolerance in various accessions of the genus *Vigna* and selected six accessions as the most tolerant accessions. These accessions were all wild accessions adapted to coastal habitats but were phylogenetically distinct from each other. This result indicated that extreme salt tolerance evolved at least four times during speciation in the genus *Vigna*. They showed diverse characteristics, especially in leaf Na^+^ concentration, indicating the acquisition of different mechanisms of salt tolerance. In addition, because of the large differences in salt tolerance between the genetically close accessions, simple genetic changes might be responsible for salt tolerance evolution. Since most of the tolerant accessions are crossable to susceptible accessions, they can be donors of salt tolerance for breeding, or would be materials for isolating tolerance genes. It would also be interesting to *de novo* domesticate the wild salt tolerant accessions. Thus, from the above results, we demonstrated that the genus *Vigna* is a valuable genetic resource of salt tolerance.

## Supporting Information

S1 Appendix Characteristics of shoot biomass, quantum yield of PSII, leaf Na^+^ concentration, and K^+^/Na^+^ ratio of the 69 accessions used in this study. (XLSX)

S1 Fig Images of the 69 accessions at 2 weeks after salt treatment. (PPTX)
