# Supplementary material for: Diversity and Evolution of Salt Tolerance in the Genus Vigna
Source: PLoS One. 2016 Oct 13;11(10):e0164711. doi: 10.1371/journal.pone.0164711 (PMC5063378; doi:10.1371/journal.pone.0164711)
Supplement: S1 Fig — (PPTX) [file pone.0164711.s002.pptx]

## Slide 1
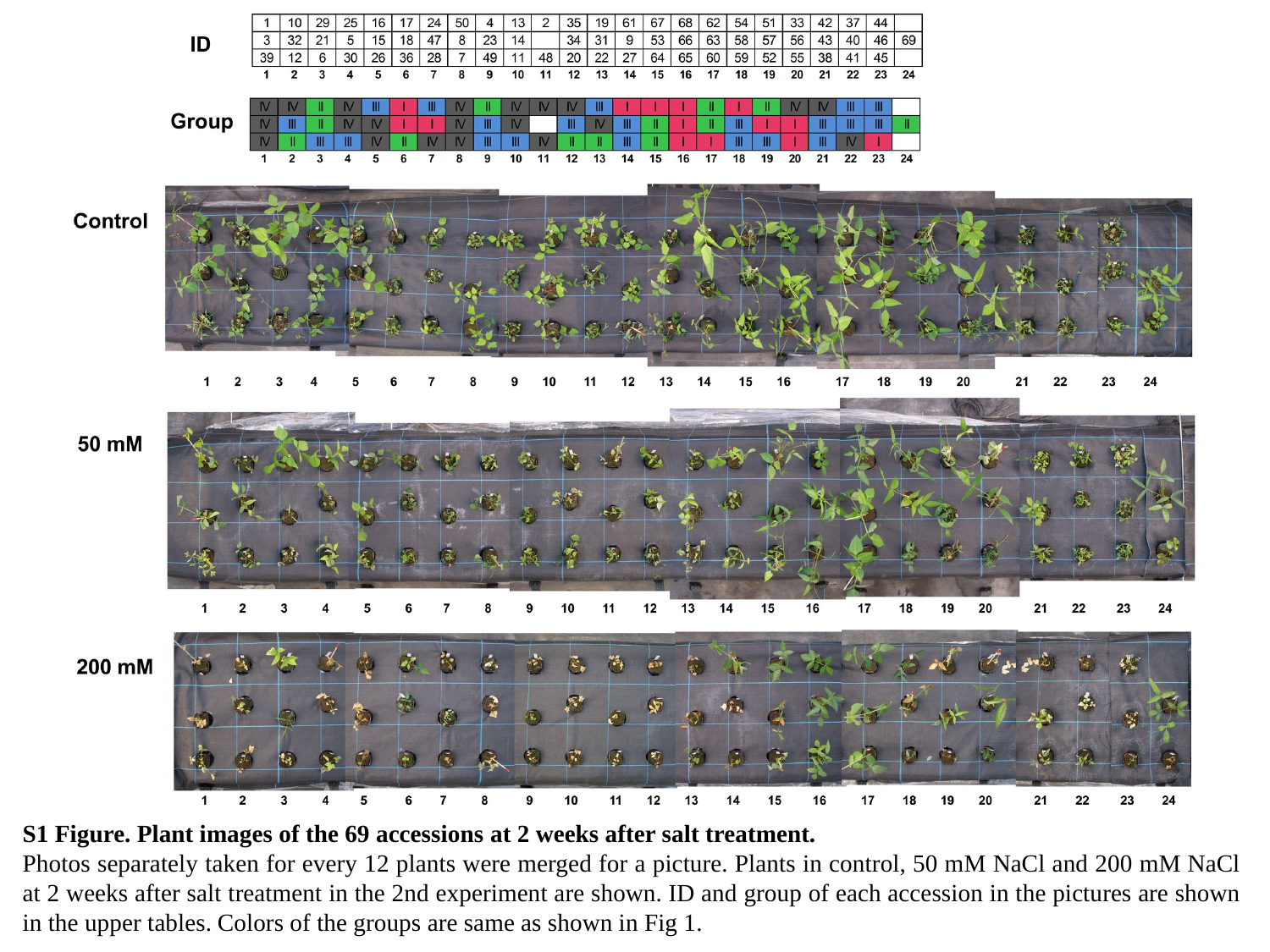

S1 Figure. Plant images of the 69 accessions at 2 weeks after salt treatment.
Photos separately taken for every 12 plants were merged for a picture. Plants in control, 50 mM NaCl and 200 mM NaCl at 2 weeks after salt treatment in the 2nd experiment are shown. ID and group of each accession in the pictures are shown in the upper tables. Colors of the groups are same as shown in Fig 1.
